# Comparative Effectiveness of Guidelines for the Management of Hyperlipidemia and Hypertension for Type 2 Diabetes Patients

**DOI:** 10.1371/journal.pone.0016170

**Published:** 2011-01-25

**Authors:** Nilay D. Shah, Jennifer Mason, Murat Kurt, Brian T. Denton, Andrew J. Schaefer, Victor M. Montori, Steven A. Smith

**Affiliations:** 1 Division of Health Care Policy and Research, Mayo Clinic, Rochester, Minnesota, United States of America; 2 Knowledge and Encounter Research Unit, Mayo Clinic, Rochester, Minnesota, United States of America; 3 Edward P. Fitts Department of Industrial and Systems Engineering, North Carolina State University, Raleigh, North Carolina, United States of America; 4 Department of Industrial Engineering, University of Pittsburgh, Pittsburgh, Pennsylvania, United States of America; 5 Division of Endocrinology, Mayo Clinic, Rochester, Minnesota, United States of America; University of Padova, Medical School, Italy

## Abstract

**Background:**

Several guidelines to reduce cardiovascular risk in diabetes patients exist in North America, Europe, and Australia. Their ability to achieve this goal efficiently is unclear.

**Methods and Findings:**

Decision analysis was used to compare the efficiency and effectiveness of international contemporary guidelines for the management of hypertension and hyperlipidemia for patients aged 40–80 with type 2 diabetes. Measures of comparative effectiveness included the expected probability of a coronary or stroke event, incremental medication costs per event, and number-needed-to-treat (NNT) to prevent an event. All guidelines are equally effective, but they differ significantly in their medication costs. The range of NNT to prevent an event was small across guidelines (6.5–7.6 for males and 6.5–7.5 for females); a larger range of differences were observed for expected cost per event avoided (ranges, $117,269–$157,186 for males and $115,999–$163,775 for females). Australian and U.S. guidelines result in the highest and lowest expected costs, respectively.

**Conclusions:**

International guidelines based on the same evidence and seeking the same goal are similar in their effectiveness; however, there are large differences in expected medication costs.

## Introduction

Clinical practice guidelines are viewed as useful tools for making care more consistent and efficient and for closing the gap between what clinicians do and what scientific evidence supports. Interest in clinical guidelines is international and has its origin in issues faced by most healthcare systems: rising healthcare costs; variation in service delivery with the presumption that at least some of this variation results in inappropriate care; and a mechanism for providing patients the best care possible.

An Institute of Medicine report on clinical guidelines recommended that information on cost implications be incorporated into guidelines; however, the report acknowledged that major methodological and practical challenges exist to implement this recommendation. [1] Over the last two decades the number of guidelines has grown internationally, and within the United States (US), but costs have generally not been considered in the US. [2] Thus, the influence of incremental guideline changes on cost is unclear. The debate on healthcare reform and methods for “bending the cost curve” motivate the importance of understanding efficiency of guidelines.

Although the burden of cardiovascular risk among diabetes patients is comparable across developed countries, there are often differences in treatment guidelines. [3] For instance, there are different thresholds for treatment decisions and medication management that do not directly reflect the available evidence, such as LDL-cholesterol levels for starting medication therapy. [4] The lack of transparency about guideline formulation has led to criticisms, and the perception that conflicts of interest may have influenced their recommendations. [2], [5], [6] Given the variation in the development and implementation of treatment guidelines internationally, it is necessary to classify the most efficient strategies. This is an increasingly important issue, given the simultaneous increase in healthcare costs and diabetes incidence. [7], [8]

We conducted a comparative effectiveness analysis of international contemporary guidelines for the management of hypertension and hyperlipidemia for patients with type 2 diabetes. In order to demonstrate the impact of changes in consensus recommendations, we include historical antecedent guidelines from the US. To compare these guidelines, we use a Markov model for type 2 diabetes to estimate measures related to primary prevention including the probability of a coronary heart disease (CHD) or stroke event, number-needed-to-treat (NNT), and expected medication cost per event avoided.

## Methods

### Markov Model

**Figure 1:**
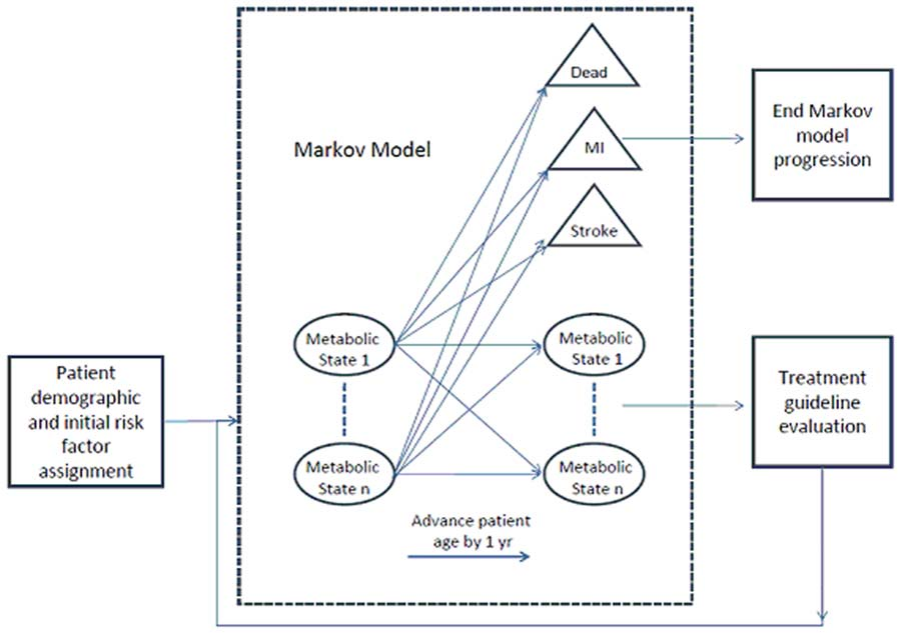
Illustration of the Markov model for treatment of type 2 diabetes. Transitions between states occur annually. Patients transition among health states defining the risk of CHD or stroke events, and treated health states based on treatment guidelines.

Our Markov model is a natural history model for type 2 diabetes patients, ages 40 to 80 years, considering three modifiable risk factors: blood glucose, hypertension, and hyperlipidemia. Figure 1 illustrates the probabilistic transitions in our model that estimates probability of an event for a given patient and treatment guideline. Appendix S1 presents additional details of the model and its assumptions. For the management of hyperlipidemia, we consider treatment with statins followed by fibrates; for hypertension management we consider treatment in the order of thiazides, angiotensin receptor blockers (ARBs)/angiotensin converting enzyme (ACE) inhibitors, beta-blockers, and calcium channel blockers (CCBs).

### Key assumptions

We assume that treatment decisions regarding the initiation of new drug therapies are revisited annually. Hyperlipidemia medications result in a proportional decrease in total cholesterol and increase in HDL levels; hypertension medications result in a proportional decrease in systolic (SBP) and diastolic blood pressure (DBP). We assume additive treatment effects for medications that influence the same risk factor. We do not consider revascularization procedures in the absence of a cardiovascular event. We also assume that the rate of adverse effects will be similar across all guidelines. We assume that patients will have perfect adherence to medications. Finally, this paper evaluates the impact of these guidelines in a US population and uses US prices for the medications. If the populations and prices are used for each of the countries and their populations, we would expect the results to be different.

### Key variables

Additional Markov model variables include patient demographics (sex, age, and time of diagnosis of diabetes) and metabolic characteristics (blood pressure, lipid, and HbA1c levels). We refer to the combination of lipids and blood pressure states with the patient's mean HbA1c as the patient's *metabolic state*. We combine this information with the patient's demographic characteristics and medication history to define the patient's CHD and stroke risk profile. We refer to this combination as the patient's *health state*.

#### Data and Model Inputs

We use the Mayo Clinic Diabetes Electronic Management System (DEMS) Data Set [9] to estimate model parameters including the transition probabilities among the metabolic states. DEMS is a diabetes data management system based on longitudinal medical records for diabetes patients at Mayo Clinic. Our cohort includes 663 patients in DEMS between 1997 and 2006 with type 2 diabetes, aged 40 to 80 during the observation period. These patients had approximately 15,000 measurements of cholesterol, blood pressure and HbA1c. These data are also used to estimate mean treatment effects for hyperlipidemia and hypertension medications.

**Figure 2:**
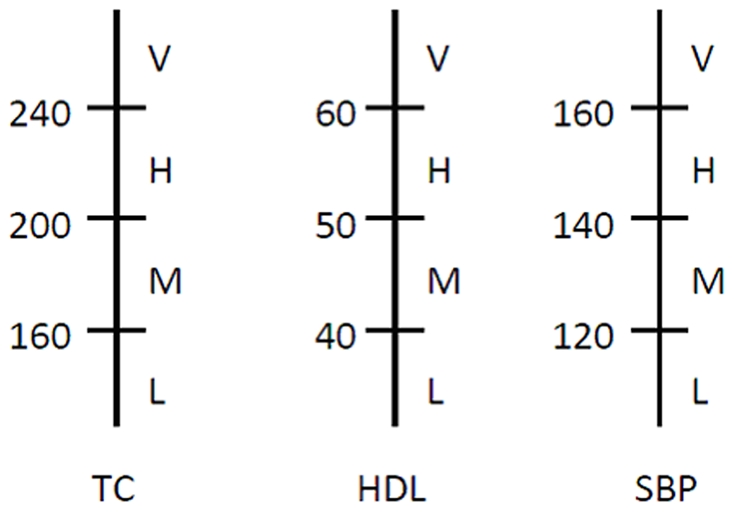
Cut-points used to define the health states for the Model (TC = Total Cholesterol; HDL = High Density Lipoprotein Cholesterol; SBP = Systolic Blood Pressure; DBP = Diastolic Blood Pressure).

Of the metabolic factors, triglycerides and HbA1c are modeled as a function of age. Due to the uncertain nature of cholesterol levels of a patient over time, we treat the progression of total cholesterol and HDL-cholesterol levels as a Markov process, each with a finite set of states (Figure 2), and then estimate LDL levels using Friedewald's equation. [10] SBP is also treated as a Markov process. A major advantage of our model is that it is developed based on 10-year observation period for each individual (except those that died during the observation period (1.7%)) and it simulates change in risk factors over time.

### Cardiovascular and stroke event rates

Probabilities for initial CHD and stroke events are calculated from the United Kingdom Prospective Diabetes Study (UKPDS) risk equations. [11], [12], [13] These probabilities depend on health states and age, and reflect the changes in the patient's health and medical treatment status. We focus on primary prevention, and therefore we do not examine the details of a patient's course after the first cardiovascular or stroke event, or death from other causes. Probability of death from other causes was estimated using data from the National Center for Health Statistics mortality rate tables. [14], [15]

### Treatment Efficacy

We use patient-level data from our cohort to estimate the effect of each of the medications on the metabolic factors. Appendix S2 presents the details of these calculations. Table 1 shows the estimated treatment effects of medications that were incorporated into the model. We assumed no interaction in the effects of medications for hypertension and hyperlipidemia.

**Table 1:** Effects of medication initiation on metabolic factors for the most commonly used medications.

		% Change		
Treatment	Total Cholesterol	HDL	Systolic Blood Pressure	Diastolic Blood Pressure
**Statins**	−14	7.3		
**Fibrates**	−3.9	4.7		
**ACE-Inhibitors/Angiotensin Receptor Blockers**			−3.7	−5.5
**Thiazides**			−5.0	−3.7
**Beta-Blockers**			−4.6	−4.2
**Calcium Channel Blockers**			−2.5	−4.8

### Costs

We conducted the analysis from the perspective of a third-party payer. We consider incremental medication costs that are directly associated with the management of hypertension and hyperlipidemia. Drug costs were obtained from the 2009 Red Book average wholesale prices. [16] For our base-case analysis, we estimated the annual medication costs using the lowest Red Book prices for simvastatin, gemfibrozil, lisinopril, metoprolol, hydrocholorothiazide, and amlodipine (Table 2). We also conducted sensitivity analyses using the highest listed Red Book prices for each medication class. Costs associated with medications for glycemic control were not considered.

**Table 2:** Annual medication costs.

Drug Class	Base-case	Upper Bound
Statins	$ 212	$ 1,258
Fibrates	$ 652	$ 1,452
Angiotensin Converting Enzyme (ACE) Inhibitors/Angiotensin II Receptor Blockers	$ 48	$ 868
Thiazides	$ 48	$ 946
Beta Blockers	$ 48	$ 145
Calcium Channel Blockers	$ 866	$ 1,031

### Guidelines

We compare guidelines using our Markov model to represent progress. The patient's risk factors evolve over time from a starting age of 40 until the time of the first event. Medication is initiated at annual intervals whenever the patient meets the guideline criteria. In contrast to other studies of cardiovascular risk management in patients with diabetes [17] our approach assumes the patient is treated incrementally, over time, as the risk evolves with age.

We considered the Adult Treatment Panel (ATP) II [18] and III [19], the Sixth [20] and Seventh [21] reports of the Joint National Committee on Prevention, Detection, Evaluation, and Treatment, of High Blood Pressure (JNC 6, JNC 7), Canadian [22], [23], European societies [24], British [25], and Australian [26], [27] guidelines for treatment of hyperlipidemia and hypertension. The guidelines are summarized in Table 3. US I represents the combination of the ATP II and JNC 6 guidelines, and US II represents the combination of ATP III and JNC 7 guidelines. We also evaluate three reference guidelines related to US guidelines: 1) initiating statins and ACE-inhibitors at the time of diagnosis with no further intensification or changes in treatment for the management of hypertension and lipids (Initiate at Diagnosis), 2) JNC 7 and the standard ATP III guidelines without considering diabetes as a risk equivalent (US III); and 3) initiating statins and ACE-inhibitors at the time of diagnosis and then following ATP III and JNC 7 (US IV). Table 3 presents a summary of the guideline combinations.

**Table 3:** Description of Guidelines (recommendations below are thresholds for treatment initiation).

Guideline	Hyperlipidemia	Hypertension
**Initiate at Diagnosis**	Initiate statins when diagnosed with diabetes with no further measurement or intensification	Initiate ACE-Inhibitors at diagnosis with no further measurement or initiation
**United States I**	ATP II: LDL≥130 mg/dL	JNC 7: SBP>130 mm Hg or DBP>85 mm Hg
**United States II**	ATP III: LDL≥100 mg/dL	JNC 7: SBP>130 mm Hg or DBP>80 mm Hg
**United States III**	ATP III: Calculate risk based on individual risk factors and treat to goal based on risk-factors (High risk: LDL≥100 mg/dL; moderate risk: LDL≥130 mg/dL; low risk: LDL≥190 mg/dL)	JNC 7: SBP>140 mm Hg or DBP>90 mm Hg
**United States IV**	Initiate statins when diagnosed with diabetes and intensify according to ATP III guidelines	Initiate ACE-Inhibitors when diagnosed with diabetes and intensify according to JNC 7 guidelines
**Canada**	LDL≥2.5 mmol/L or LR≥4	SBP>130 mm Hg or DBP>80 mm Hg
**European Societies**	LDL≥2.5 mmol/L or TC≥4.5 mmol/L	SBP>130 mm Hg or DBP>80 mm Hg
**Joint British Societies**	LDL≥2.0 mmol/L or TC≥4 mmol/L	SBP>130 mm Hg or DBP>80 mm Hg
**Australia**	LDL≥2.5 mmol/L or TC≥ mmol/L or HDL<1 mmol/L	SBP>130 mm Hg or DBP>80 mm Hg

In addition to the above guidelines we also evaluated the impact of intensive blood pressure control as recently reported in the ACCORD study. [28] In this case lipids were managed using the standard ATP III guideline and hypertension was managed to a target SBP of 120 mm/Hg. We did not consider the case of combination lipid-lowering therapy as considered in ACCORD [29]; patients received combination treatment as recommended by the ATP III guidelines.

### Outcome Measures

We evaluated the performance of the guidelines based on implications for patient outcomes, clinical policy, and health policy. Patient outcomes are measured by the probability of CHD or stroke event. Clinical policy measures include the NNT for a lifetime (represented here by the span of time from age 40 until death or age 80), and the number of events avoided per 1,000 patients treated. Health policy implications are measured by expected medication costs per event avoided. We use a 3% annual discount rate for costs. [30]

### Role of the funding source

The Agency for Healthcare Research and Quality (AHRQ) and the National Science Foundation (NSF) had no role in the study design, conduct, analysis, or manuscript preparation for this study.

## Results

### Base Case

**Figure 3:**
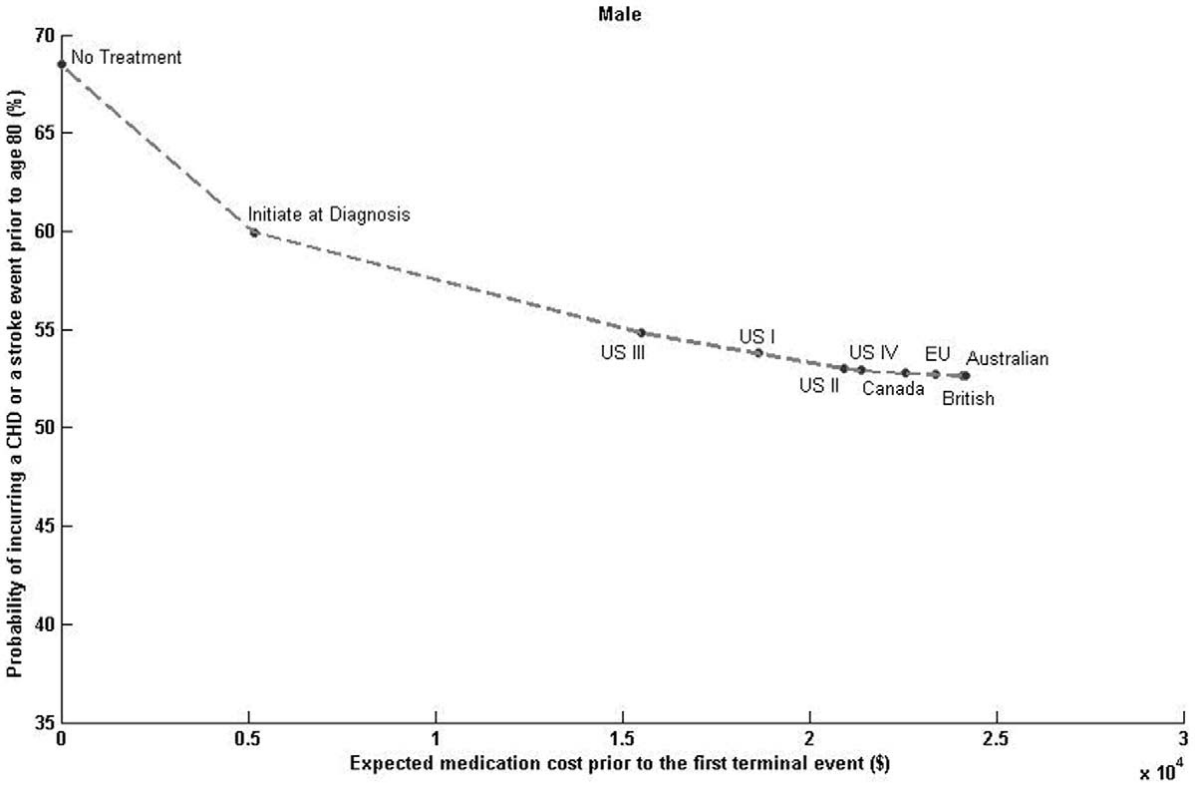
Efficient Frontier for Treatment Guidelines for Males.

**Figure 4:**
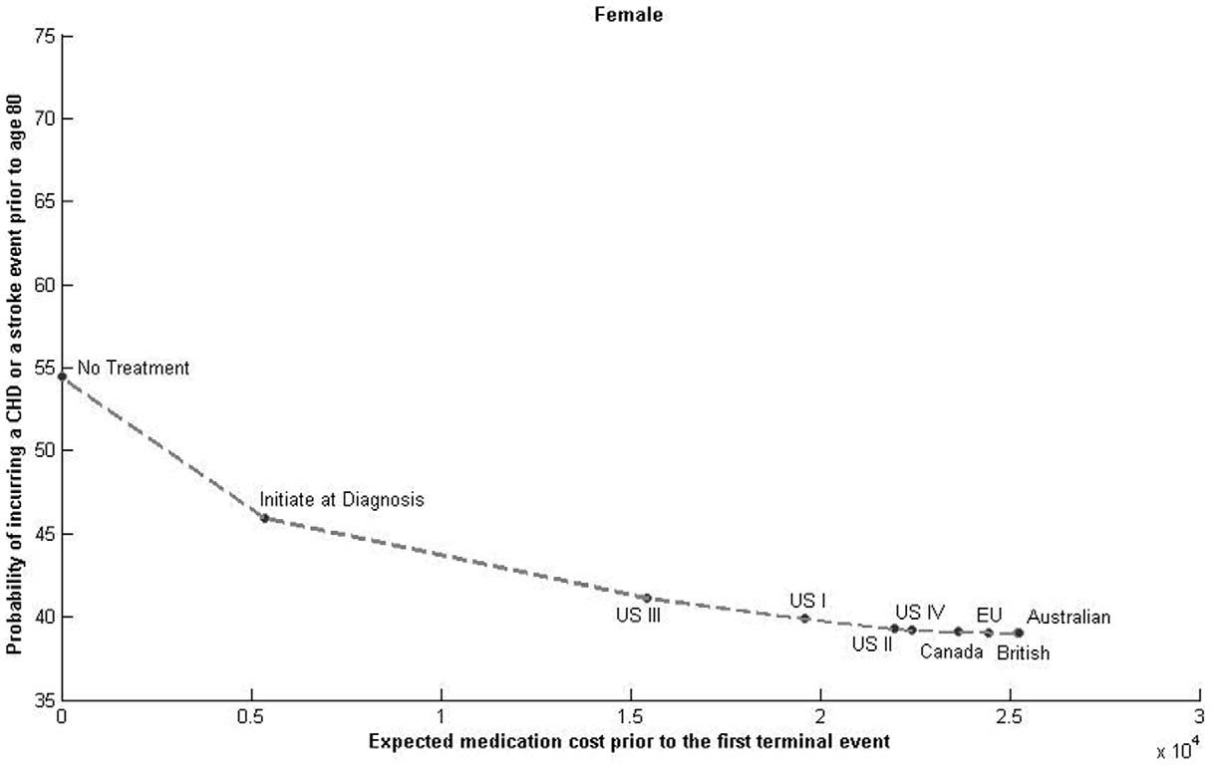
Treatment Guidelines for Females.

The time horizon spanned ages 40 to 80 years. We evaluated the guidelines for Caucasian, non-smoking patients of both sexes, diagnosed with type 2 diabetes at age 40 and with no prior history of CHD, stroke or cardiac disorders. The proportion of patients in each health state at age 40 was based on our study cohort. Figures 3 and 4 present the probability of an event, and the expected medication cost for each of the guideline combinations using a discount factor of 3%.

Assuming no treatment, the probability of an event between ages 40 to 80 was 68.5 percent for male patients, and 54.5 percent for female patients. Using US III decreased the probability of an event to 54.8 percent for males and 41.2 percent for females. For both sexes, there were some differences in the probability of an event across the international guidelines; however, these differences were small. The maximum difference in the probability of having an event across guidelines was 2.1 percent for males and 2.2 percent for females. The Australian guideline combination had the lowest probability of incurring an event (52.7% for males and 39.0% for females). The guideline with the highest probability of an event was US III (54.8% for males and 41.2% for females).

From a clinical policy perspective, it is important to consider the role of both hypertension and hyperlipidemia guidelines since their collective goal is primary prevention. We observed small variation in the NNT across guidelines (Table 4). Australian, British, European and Canadian guidelines resulted in the lowest NNT for males and females; the highest NNT was for the risk blind initiation at diagnosis of statins and ACE inhibitors with no further management of hypertension or lipids (NNT = 14.4 for males and 11.7 for females). Among the US guidelines, the NNT was highest for the US III guidelines (NNT = 7.6 for males and 7.5 for females). The changes from US I to US II for diabetes required an increased intensification of lipid and blood pressure control. These incremental changes in guidelines did not have a significant impact in changing the absolute risk for primary prevention.

**Table 4:** Impact of Guidelines.

Guidelines	Guideline Impact			Guideline Impact		
	Males			Females		
	Number Needed To Treat	Number of Events Avoided per 1,000 Treated	Medication Costs per Event Avoided	Number Needed To Treat	Number of Events Avoided per 1,000 Treated	Medication Costs per Event Avoided
United States I	7.0	142.6	$129,428	6.9	145.6	$134,655
United States II	6.7	150.4	$139,204	6.6	151.8	$144,773
United States III	7.6	132.3	$117,269	7.5	133.0	$115,999
United States IV	6.6	151.4	$141,185	6.6	152.5	$147,011
Canada	6.6	152.8	$147,705	6.5	153.6	$153,952
European Societies	6.5	153.3	$152,385	6.5	153.9	$158,784
Joint Bristish Societies	6.5	153.8	$156,817	6.5	154.1	$163,488
Australian	6.5	153.9	$157,186	6.5	154.3	$163,775
Statin + ACE Inhibitor with no guideline	14.4	81.0	$63,708	11.7	70.7	$75,886

*United States III - Assumes diabetes as a cardiovascular risk equivalent.

**United States IV - Immediate initiation of statins and ACE Inhibitors after diagnosis of diabetes.

From a health policy perspective, there were differences in the expected medication costs across the guidelines and between sexes. With the base-case (lower) costs of medication, for males, the expected medication costs varied between $15,509 (US III) and $24,186 (Australian), and for females between $15,433 (US III) to $25,267 (Australian). For the highest estimate of medication costs (specifically, branded medications), expected medication costs varied between $57,085 (US III) and $87,961 (Australian) for males, and between $57,792 (US III) and $91,691 (Australian) for females.

We estimated the policy implications for each of the guidelines. The US III guideline had the lowest expected medication costs per event avoided per 1,000 diabetes patients ($117,269 for males; $115,999 for females). The Australian guideline combination was the most expensive. The incremental expected medication costs per event avoided (per 1,000 diabetes patients) when going from US I to US II was $335,310 for males and $384,175 for females.

Evaluation of guidelines with intensive blood-pressure management, as reported by ACCORD, showed that the impact of intensive treatment was small compared to US II guidelines (NNT = 222 for males and NNT = 258 for females). In addition, the incremental cost increase per event avoided per 1,000 diabetes patients was $1.4 million for males and $1.7 million for females.

## Discussion

### Key findings

Our results suggest that the various treatment guidelines for hyperlipidemia and hypertension for diabetes patients have become increasingly similar in their effectiveness in recent years. However, there are differences in lifetime medication costs for the management of hyperlipidemia and hypertension.

A surprising finding was that the US guidelines are generally more efficient compared to other published guidelines. This is largely due to the lower lipid targets being pursued by the other countries/regions. It is important to note that there is little variation among hypertension guidelines, and thus the differences in results are largely driven by differences in lipid management.

Sensitivity analysis using a range of US costs for medications did not change the relative efficiency. Australian guidelines have the highest and US guidelines the lowest expected medication costs per event avoided. Changes in treatment guidelines from ATP II to ATP III, and from JNC 6 to JNC 7, simultaneously increased the expected costs per event avoided. More importantly, the benefits of these changes were minimal as evidenced by the large NNT. Figures 3 and 4 show that the relative order of efficiency of contemporary guidelines is generally the same for males and females.

We found that the ATP III guidelines that consider diabetes as a cardiovascular risk equivalent decreased the probability of having an event by 1.8 percent for males and by 1.9 percent for females compared to US III. Among US guidelines, US IV has the lowest probability of an event for both males and females; however, this strategy also has the highest cost. Simulation of intensive treatment as suggested by ACCORD showed relatively small benefit for significantly higher costs as suggested by the results of the trial. [28] Sensitivity analysis with higher medication costs did not change these results. However, these analyses point out the large differences in costs that can be incurred based on the choice of medications. The routine use of branded drugs can increase the lifetime costs of medications by almost four fold for both males ($79,915 compared to $20,931) and females ($83,558 compared to $21,970).

### Implications

The results of our analyses suggest that there are significant differences in patient outcomes, clinical policy, and health policy measures. According to our model if one were to treat 1 million males with newly diagnosed type 2 diabetes according to the most costly (Australian) and current US guidelines (US II), there would be significant differences in societal costs with minimal differences in outcomes. The Australian guidelines would be expected to incur an additional expected medication cost of $3.3 trillion dollars and lead to 2,518 fewer people experiencing a cardiovascular event.

A previous evaluation comparing guidelines for the management of hyperlipidemia found that the New Zealand guideline was the most efficient while the ATP III guidelines were least efficient. [31] The ATP III guidelines would have treated twice as many individuals as the New Zealand guidelines; however, it would have prevented a similar number of deaths. Interestingly, in our analysis, all international guidelines would lead to fewer events, but they would also be more costly than the US guidelines. These differences may be due to the recent changes in international guidelines where diabetes is considered a cardiovascular risk equivalent. In our analysis, we did not separate out the New Zealand guidelines since they are the same as the Australian guidelines for diabetes patients. Recent results by Timbie et al. suggest that, even among diabetes patients, a risk-targeted approach may lead to greater benefits and fewer harms. [17] It is important for policy makers to consider these tradeoffs that may be incorporated implicitly within guidelines as health care spending becomes an increasingly finite resource that needs to be managed more efficiently.

### Limitations and strengths

There are several limitations associated with our analyses. Our Markov model is based on point estimates of transition probabilities among metabolic states, cost parameters and literature driven cardiovascular event probabilities, and each estimate is subject to statistical variance. Another potential source of error is model uncertainty. An example of model uncertainty is our assumption that costs for non-pharmaceutical medical care for the management of cardiovascular disease in different countries for type 2 diabetes patients are the same. A further limitation is that we did not consider medication adverse effects, non-adherence and discontinuation. We partially addressed this through estimating the medication effectiveness from observational data. We assume that all other medical costs for patients will be equal across the guidelines and thus, focus our comparison on medication costs.

We estimate risk reduction of initiating medications based on the UKPDS risk equation by incorporating the changes in laboratory values after initiating medication. However, the Heart Protection Study suggests that statins used at fixed doses without monitoring for achievement of a lipid goal and without dose titration could reduce CV risk by 25% regardless of the extent to which that statin dose reduced lipid levels. [32] This could influence the relative efficiency of guidelines.

Our model has several advantages. We use longitudinal patient-level observational data to calibrate a natural history model of hyperlipidemia and hypertension progression over time. In contrast to previous studies we evaluate guidelines based on their application to treatment over the course of a patient's lifetime as their CHD and stroke risk evolves. [17] Although, our data is restricted to a single health care institution, it provides a data set which is rich enough in clinical information that is necessary for our model. Finally, we use estimates of the effectiveness of medications in the real world as opposed to previously published models which use data from clinical trials that may overstate effectiveness due the selection bias and the rigor associated with clinical trials. [33],[34] On the other hand, we did not include unintended consequences or side effects that could have decreased the efficacy of the intervention in reducing CHD or stroke events (e.g., intensive blood pressure control leading to hypotension and stroke).

### Conclusion

Hyperlipidemia and hypertension guidelines have a large societal impact as they treat a large portion of the population and result in high costs. Used in combination, hyperlipidemia and hypertension medications can significantly lower the probability of a cardiovascular event or stroke in the diabetes population. There are significant differences in medication cost for patients treated under different international guidelines; however, the reduction in probability of events is similar across guidelines. The recent results from the ACCORD and INVEST trials raise questions about the impact of intensive hypertension management. [35],[28] Policymakers implementing healthcare reform must evaluate the current guidelines and the associated tradeoffs in benefits, risks, and costs. A more risk targeted approach for the management of cardiovascular risk for diabetes patients has the potential to have large benefits while reducing costs.

## Supporting Information

Appendix S1 Markov Model.
(DOC)

Appendix S2 Estimation of Effectiveness of Treatment Initiation.
(DOC)
