# Supplementary material for: Comparative Effectiveness of Guidelines for the Management of Hyperlipidemia and Hypertension for Type 2 Diabetes Patients
Source: PLoS One. 2011 Jan 25;6(1):e16170. doi: 10.1371/journal.pone.0016170 (PMC3026790; doi:10.1371/journal.pone.0016170)
Supplement: Appendix S1 — Markov Model. (DOC) [file pone.0016170.s001.doc]

**Appendix S1. Markov Model**

We model the progression of the disease as a Markov model, where patients transition among health states until they enter an *absorbing state*, which comprises CHD or stroke events, or death from all other causes. Patients begin at age 40 after being diagnosed with the disease and follow a stochastic progression of the disease (defined by the Markov model) prior to the occurrence of the first event or the age of 80. The use of the Markov model to evaluate treatment guidelines is illustrated in Figure 1.

We assume the patient may initiate new drug therapies annually. Decisions about the initiation of new therapies for cholesterol and blood pressure control are governed by the treatment policies in Table 3. Each guideline defines recommended thresholds for the levels of metabolic factors and/or for the long-term CHD risk to initiate a new treatment. At any decision stage, for a given guideline, the patient’s health state and current treatment regimen, are used to determine if any treatment should be initiated based on the guideline.

The set of treatable metabolic factors are *B* = {1, 2, 3} (1: for total cholesterol, 2: for HDL, 3: for blood pressure). Each of these may take on 4 levels: low (L), medium (M), high (H), very high (VH) determined by 25 and 75 percentile ranking that aligned well clinically relevant cut points for decision making for the guidelines evaluated. The patient’s metabolic state in each year is *h* = (*h1*, *h2*, *h3*). The current treatment regimen at time stage *t* is denoted by *mt* = (*m1*,*t*,*m2,t*), where *m1,t* is for cholesterol and *m2,t* is for the blood pressure treatment state. The total health state of the patient is the combination of health state and treatment regimen, (*h*,*mt*). The decisions recommended by the guidelines for treating cholesterol and blood pressure for a patient in state (*h*,*mt*) at time stage *t* are denoted by a 1×2 vector dmt h , where the first and second components of this vector refer to cholesterol and blood pressure treatment decisions, respectively.

We have two types of transition probabilities in our model: health state transition probabilities, and probabilities for the transitions from health states to event states. We denote the transition probability between any two metabolic states *h* = (*h1*, *h2*, *h3*) and *h’* = (*h’1*, *h’2*, *h’3*) by *p(h’|h)*. This is estimated from our study cohort as the probability of observing the total cholesterol in state *h’1*, HDL in state *h’2*, and blood pressure in state *h’3* given they are observed in states *h1*, *h2* and *h3*, respectively.

We estimate the probabilities of entering absorbing states based on the patient’s state using the UKPDS model. The survival probabilities and guideline based treatment actions are used to estimate the expected time to first event and medication costs across all possible realized scenarios that are defined by the Markov process. These are computed exactly using backwards induction over every possible state of the Markov process, (*h*,*mt*), at each age(29). The reported expected time to first event and medications costs are the weighted expectations across all states using the relative proportion of patients in each health state in our study cohort.
