# Supplementary material for: Comparative Effectiveness of Guidelines for the Management of Hyperlipidemia and Hypertension for Type 2 Diabetes Patients
Source: PLoS One. 2011 Jan 25;6(1):e16170. doi: 10.1371/journal.pone.0016170 (PMC3026790; doi:10.1371/journal.pone.0016170)
Supplement: Appendix S2 — Estimation of Effectiveness of Treatment Initiation. (DOC) [file pone.0016170.s002.doc]

**Appendix S2. Estimation of Effectiveness of Treatment Initiation**

We first estimate coefficients that represent the medication effects in patient level by taking the percent difference between the average levels of the affected metabolic factor in the second and first halves of the year following the initiation of the therapy. By averaging these coefficients over the cohort of the study we estimate the expected effects of the therapies on targeted metabolic factors. For blood pressure treatment, we consider a randomized use of ACE inhibitors and ARBs. By using our data set, we estimate the effects of such a randomized use on SBP and DBP by taking the weighted average of the individual effects of these therapies, where the weights are relative frequencies of the use of these drugs in the study cohort.
